# Bioactive Edible Sodium Alginate Films Incorporated with Tannic Acid as Antimicrobial and Antioxidative Food Packaging

**DOI:** 10.3390/foods11193044

**Published:** 2022-09-30

**Authors:** Han Li, Chen Liu, Jingrong Sun, Shanshan Lv

**Affiliations:** College of Forestry, Northwest A&F University, Xianyang 712100, China

**Keywords:** UV-shielding, antioxidant activity, antibacterial activity, films

## Abstract

Currently, biodegradable and functional food packaging materials have attracted more and more attention due to their potential advantages. Biopolymers are one of the promising materials used to produce biodegradable food packaging films, and sodium alginate (SA) is one of the most used polysaccharides. In this work, we explored a novel edible sodium alginate (SA)/tannic acid (TA) film as biodegradable active food packaging material. The impact of TA concentration on the UV light blocking ability, transparency, water vapor barrier ability, mechanical strength, antioxidant, and antimicrobial activity of the SA-TA films was comprehensively investigated. Fourier transform infrared spectroscopy results revealed that strong hydrogen bonding was the main intermolecular interaction between SA and TA. As TA concentration in the films increased, the water vapor permeability (WVP) decreased from 1.24 × 10^−6^ to 0.54 × 10^−6^ g/m/h/Pa, the DPPH radical scavenging activity increased from 0.008% to 89.02%. Moreover, the incorporation of TA effectively blocked UV light and elevated antimicrobial activity against *Escherichia coli*. Overall, the SA films with TA exhibited better water vapor barrier ability, remarkable UV-light barrier ability and antioxidant activity while showing a slight decrease in light transmittance. These results indicated the potential application of TA as a functional additive agent for developing multifunctional food packaging materials.

## 1. Introduction

Considering the concerns of environmental pollution caused by synthetic plastics and food safety demands from consumers, biodegradable and recyclable food packaging materials from natural biopolymers such as polysaccharides, proteins, and polylactides are becoming increasingly popular among researchers and industry [1,2]. Among these biodegradable polymers, polysaccharides are considered to be an alternative to traditional plastic packaging materials. Particularly, sodium alginate, starch, chitosan, nanocellulose, and pectin have been utilized as food packaging materials frequently because their good biodegradability and excellent edible and film-forming properties.

Sodium alginate (SA) is a linear and biodegradable polyuronic acid that consists of α-L-guluronate and β-D-mannuronic [3]. With good oxygen barrier properties, low toxicity, good compatibility and biodegradability, low cost, and prominent film-forming ability, SA has been widely used in the pharmaceutical and food industries. However, the application of SA films is still limited by their low water barrier ability, poor ultraviolet light barrier ability, lack of antioxidant activity and antimicrobial activity. Thus, an effective approach to improve these properties is needed. In recent years, incorporation of natural bioactive additives into food packaging films to improve the functional performances of packaging films has received considerable attention [4]. Currently, many natural extracts, such as plant essential oils, tea polyphenols, cinnamaldehyde, carvacrol, tannic acid, citric acid, and flavonoids, have been mixed with SA to endow the films with more functionalities [3,4,5].

Tannic acid (TA) is an inexpensive polyphenol compound due to its abundant resource from plants. The large amount of phenolic hydroxyl groups on the molecular chains of TA endows it multiple bioactive attributes. For example, excellent antioxidant activity, anti-ultraviolet, and antimicrobial capacity have been widely reported for TA [5,6,7]. Additionally, TA can interact with other biopolymers, such as polysaccharides, alkaloids and proteins, through cross-linking and intermolecular hydrogen bonding, consequently improve the mechanical strength, barrier properties and antioxidant activity. Previous study found that the addition of TA into cellulose nanofibrils films (CNF) can effectively enhance the UV-shielding and antioxidant ability of the CNF films [8]. The gelatin films designed with TA and cellulose nanocrystals also exhibited improved UV barrier, antioxidant activity, water vapor barrier, and antibacterial effects against *Escherichia coli* and *Staphylococcus aureus* [9]. Furthermore, the Food and Drug Administration (FDA) generally recognizes TA as safe (GRAS), meaning it is safe for direct use in food packaging. Therefore, TA is considered a potential additive to fabricate biodegradable and bioactive film for food packaging. Up to now, some studies on packaging films incorporated with tea polyphenol have been reported [8,10,11,12]. As far as we know, however, limited research has been performed on functional SA films with TA addition.

It is important for food packaging films to have a water vapor barrier capability. A better water vapor barrier can prevent moisture transfer from food products to the surrounding environment, thereby slowing down the water loss of food products (especially for vegetables and fruits) and prolong their shelf life. Generally, some food products are sensitive to UV light and oxygen. When these products are exposed to ultraviolet light and/or atmosphere with high oxygen, various negative effects such as discoloration, flavor and nutrient loss can be caused. Therefore, it is useful and important to develop UV-blocking and antioxidant-activated packaging films. Furthermore, antimicrobial activity is desirable and preferable for food packaging materials so that food products can remain fresh for a longer period.

Thus, in this work, a multifunctional edible film based on SA was prepared for bioactive food packaging by using the solution casting method, and TA was used as a bioactive additive to enhance the SA films’ performance and functionalities. The impact of TA concentration on the physicochemical properties, thermal stability, light barrier capacity, antimicrobial activity, and antioxidant ability of SA films were investigated. By incorporating bioactive polyphenol compounds, a simple strategy for designing edible food packaging films is demonstrated in this work.

## 2. Materials and Methods

### 2.1. Materials

Tannic acid (TA, AR grade) and sodium alginate (SA, AR grade) with 90% purity were provided by Macklin Biochemical Co., Ltd. (Shanghai, China). Glycerol was supplied by Chengdu Kelong Chemical Co., Ltd. (Chengdu, China). 2, 2-diphenyl-1-picrylhydrazyl (DPPH), Luria–Bertani (LB) broth and LB agar were obtained from Solarbio Science & Technology Co., Ltd. (Beijing, China). All other reagents were analytical grade and the experiments were carried out with deionized water.

### 2.2. Fabrication of Edible Sodium Alginate Films

Casting method was used to fabricate the SA films according to previous study [13]. First, the SA and TA powder were dissolved in deionized water and magnetically stirred for 6 h to obtain SA and TA storage solutions. Subsequently, the SA and TA storage solutions were mixed to obtain film-forming solutions (FFS), the detailed composition of the film forming solutions is listed in Table 1. Then, 20% glycerol (based on SA content) was added as plasticizer. The FFS was stirred continuously for another 2 h to obtain homogenous mixture. Finally, the FFS was degassed and poured in acrylic glass plate (10 × 10 cm^2^) and dried for 12 h at 40 °C before peeling-off. Then the films were conditioned at 25 °C and 50 ± 1% relative humidity for at least 48 h prior to further characterization. For convenience, the films were named SA-TA0, SA-TA1, SA-TA2, SA-TA3, SA-TA4, SA-TA5 groups, respectively.

### 2.3. Characterization

#### 2.3.1. Fourier Transform Infrared Spectroscopy (FTIR)

Fourier transform infrared spectroscopy (FTIR, Bruker Tensor II, Bremen, Germany) was carried out to analyze the films’ chemical structure. The spectra were obtained in the range of 4000 cm^−1^ to 400 cm^−1^ with a resolution of 4 cm^−1^.

#### 2.3.2. Light Transmission and Transparency

The light transmission of the films was determined by a UV-Vis spectrometer (TU-1901, Purkinje General, Beijing, China) at a wavelength range of 200–800 nm. According to the light transmittance at this range, the transparency and UV barrier performance of the films were obtained. The films’ transparency was calculated as follows:

$$\mathrm{Transparency}=\frac{\log T_{600}}{L}$$

where *T*_600_ represents the transmittance at 600 nm and *L* (mm) is the film’s thickness. Each sample was measured in triplicate.

#### 2.3.3. Color Parameters

The films’ color parameters were measured using a colorimeter (CS-820, CHN Spec, Hangzhou, China). L* (brightness), a* (green/red) and b* (yellow/blue) color values were obtained and the control film was used as the standard. Each sample was measured at least three times and three samples were measured for each replication. The films’ total color difference (ΔE), whiteness index (WI), and chroma (C*) were calculated as follows:

$$\Delta E=\sqrt{{\left(\Delta L^{*}\right)}^{2}+{\left(\Delta a^{*}\right)}^{2}+{\left(\Delta b^{*}\right)}^{2}}$$

$$\mathrm{WI}=100-\sqrt{{\left(100-L\right)}^{2}+{a^{*}}^{2}+{b^{*}}^{2}}$$

$$C^{*}=\sqrt{{a^{*}}^{2}+{b^{*}}^{2}}$$

where ΔL*, Δa*, and Δb* represent the color parameter’s difference between sample and control.

#### 2.3.4. Water Vapor Permeability (WVP)

Water vapor permeability (WVP) of films was measured according to ASTM E96 with some modifications. First, 2.0 g of anhydrous calcium chloride (CaCl_2_) was added into each test cup (internal diameter of 20 mm and a depth of 25 mm). Then the test cups were sealed with films and placed into a desiccator with a relative humidity of 75% (generated by saturated sodium chloride solution) at 25 °C for 7 days. Thereafter, the test cups were weighted every 12 h. Three replicates were taken for each sample, and the following equation was used to calculate the film’s WVP (g m^−1^ h^−1^ Pa^−1^):

$$\mathrm{WVP}=\frac{\Delta w\times L}{t\times A\times \Delta P}$$

where *L* is the film’s average thickness (m), Δ*w* is the test cup’s weight change (g), *A* is the area of films exposed to moisture (m^2^), *t* is the measuring time (h), Δ*P* is the water pressure difference on both sides of the film.

#### 2.3.5. Tensile Strengths (TS)

Tensile strengths (TS) of films were characterized using an electronic stripping tester (BLD-200N, Labthink, Jinan, China). The films were cut into 80 × 15 mm rectangular strips. The initial grip separation and cross-head speed were set at 50 mm and 100 mm/min, respectively. For each film, at least five replicate measurements were performed to obtain an average value.

#### 2.3.6. X-ray Diffraction (XRD)

X-ray diffraction (XRD) was performed on a commercial X-ray Cu Ka radiation diffractometer (Ultima IV, Rigaku, Tokyo, Japan) with a rate of 5°/min from 5° to 80° at 40 kV and 40 mA.

#### 2.3.7. Thermogravimetric Analysis (TGA)

A thermogravimetric instrument (TG 209F1, NETZSCH, Waldkraiburg, Germany) was applied to measure the thermal stability of films from 30 to 600 °C with a heating rate of 10 °C/min under nitrogen conditions.

### 2.4. Antioxidant Properties

The films’ antioxidant activity was evaluated by DPPH radical scavenging activity according to a previous method [13]. Briefly, 25 mg film was dissolved in 5 mL deionized water to get a sample solution. Then, 0.1 mL sample solution and 3.9 mL DPPH ethanol solution (0.1 mM) were mixed vigorously and incubated in darkness at room temperature for 30 min. Afterward, the mixture solution’s absorbance at 517 nm was characterized on a UV-1200 spectrophotometer (MAPADA, Shanghai, China). The experiment was repeated three times and the control was carried out using the similar procedure. DPPH radical scavenging activity was calculated according to the following equation:

$$\mathrm{DPPH}\;\mathrm{radical}\;\mathrm{scavening}\;\mathrm{activity}\;\left(\%\right)=\frac{A_{control}-A_{sample}}{A_{control}}\times 100$$

where *A_control_* and *A_sample_* represent the absorbance of control and sample, respectively.

### 2.5. Antimicrobial Activity

The films’ antimicrobial activity was evaluated against *Escherichia coli* (*E. coli*, DH5alpha) and *Staphylococcus Aureus* (*S. aureus*, ATCC6538) via disc diffusion method by assessing the inhibition zone size (mm). Briefly, the bacterial strain was first incubated at 37 °C for 24 h to obtain bacterial suspension. The suspension was adjusted to a fixed concentration (OD600 = 1.0) and diluted for 100 folds. Then, 100 μL of the diluted suspension was inoculated on solidified Luria–Bertani (LB) agar plate. Afterward, the film samples were cut into 6 mm discs and sterilized by ethanol and UV lighting. The sterile film discs were attached to the inoculated LB agar and incubated at 37 °C for 24 h. Finally, the inhibition zones were used to evaluate the films’ antimicrobial activity.

### 2.6. Statistical Analysis

Statistical analysis was calculated by one-way analysis of variance (ANOVA) and Tukey test was used to assess the significance of each mean value by using the OriginPro 2021 (OriginLab, Northampton, MA, USA). All the data were expressed as mean ± standard deviation (SD). Difference was considered to be statistically significant if *p* < 0.05. All experiments were performed in at least three replications.

## 3. Results and Discussion

### 3.1. Functional Groups and Chemical Bonds Characterization

**Figure 1:**
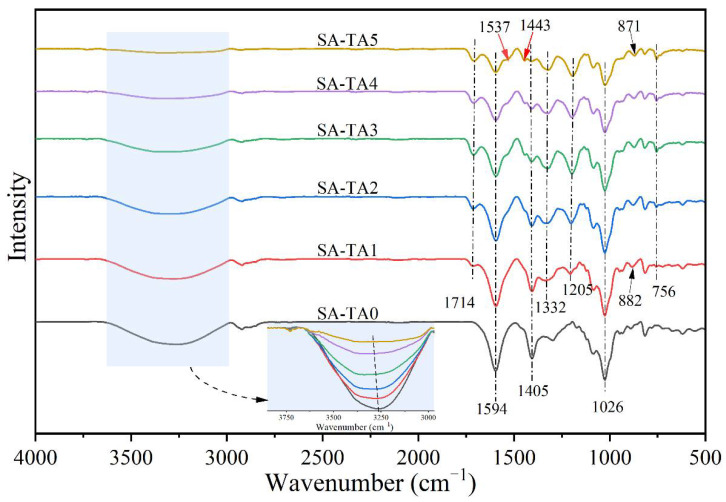
FTIR spectra of neat SA and SA-TA films.

The ATR-FTIR spectra in Figure 1 provide chemical structural insights of the SA/TA films. The SA-TA0 film exhibited a broad band in the range between 3600 cm^−1^ and 3000 cm^−1^ corresponding to -OH stretching and hydrogen bonding [14]. The absorption bands between 3600 cm^−1^ and 3000 cm^−1^ became broader and shifted to higher wavenumbers, and the intensity in this range decreased with the TA addition. These confirm the formation of strong hydrogen bonding between SA and TA [15]. The bands at 2920 cm^−1^–2800 cm^−1^ and 1026 cm^−1^ belonged to the stretching vibrations of -CH groups and C–O–C linkages in the glycosidic bonds [16]. The peak attributed to the -CH groups was attenuated by the presence of TA. The intense absorption bands at 1594 cm^−1^ and 1405 cm^−1^ were respectively assigned to the asymmetric and symmetric stretching vibrations of carboxylate ion (-COO^−^) in the SA [14,17]. These characteristic bands were attenuated when the TA was included in the SA matrix, which was resulted from the decrease proportion of SA in the films.

**Figure 2:**
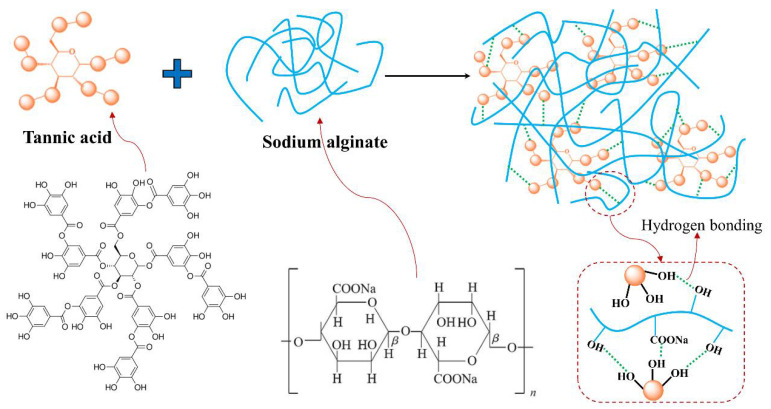
Illustration of intermolecular interaction between TA and SA.

In the case of SA-TA films, the new absorption band at 1714 cm^−1^ was associated with C=O stretching of the ester in TA, and those at 1537 cm^−1^ and 1443 cm^−1^ were attributed to the aromatic skeletal (C=C-C) vibration, with that at 1205 cm^−1^ representing typical C–O stretching vibration of polyols from polyphenol, and at 756 cm^−1^ belonging to the bending vibration of C–H in benzene rings [6,18,19,20,21,22]. With the increasing of TA concentration, the C=O and C–O vibration bands shifted from 1714 cm^−1^ to 1710 cm^−1^ and 1205 cm^−1^ to 1194 cm^−1^, respectively. This suggested stronger hydrogen bonding between SA and TA was formed [23,24]. The bands at 1332 cm^−1^ and 882 cm^−1^ were linked to phenol group and C–H bonds in the benzene rings [20,25]. From the SA-TA1 to SA-TA5 film, the band attributed to phenol group shifted from 1332 cm^−1^ to 1321 cm^−1^, suggesting the formation of hydrogen bonding between SA and TA again. All the slight shift of functional groups vibrations indicated the formation of hydrogen bonds between SA and TA, confirming the existence of intermolecular interactions between the components of the films [26]. In general, the bands position of the SA-TA composite did not change obviously, and the main change was on the peak intensity due to the hydrogen bonds formation between SA and TA, which was similar with the previous studies [27,28]. The illustration of intermolecular interactions between SA and TA is shown in Figure 2.

### 3.2. Light Transmission and Transparency

**Figure 3:**
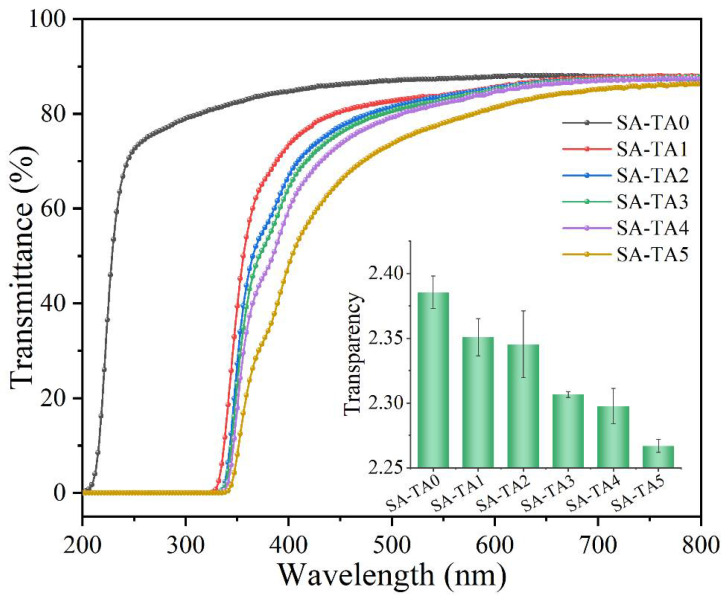
UV-vis light transmittance of neat SA and SA-TA films (insertion is the transparency of all films at 600 nm).

**Figure 4:**
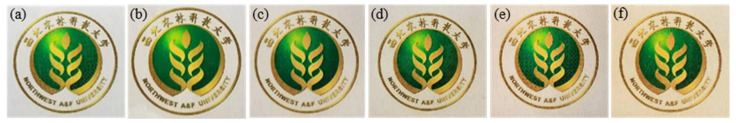
Digital photographs of the films, (**a**) SA-TA 0, (**b**) SA-TA 1, (**c**) SA-TA 2, (**d**) SA-TA 3, (**e**) SA-TA 4, (**f**) SA-TA 5.

Transparency and light transmission of films in the food packaging field are of importance, especially for food surface application [29]. Generally, light transmittance can cause of oxidation and degradation of lipids, pigments, and nutrients [26,30], which will shorten the shelf life of food products. Therefore, lower transmittance is a desirable attribute for the films applied over light-sensitive foods. The light transmittance of SA films with different TA concentrations was measured in the wavelength range of 200–800 nm (Figure 3). The transmittance of SA-TA films in the visible range of 320–800 nm was decreased by the incorporation of TA, and the films’ transparency values slightly decreased from 2.38 to 2.27. These indicated that the incorporation of TA had less influence on the SA-TA films’ transparency, which was also agreed with the digital photographs shown in Figure 4. In the range of 320–400 nm, the light transmittance of all films with TA decreased sharply as the wavelength decreased, suggesting the incorporation of TA could improve the UV-A light barrier property of SA-TA films. What is more, the light transmittance decreased significantly in this region as the TA concentration increased, which implied that high concentration of TA could absorb more UV-A light and improve the light barrier much better. In the range of 200–320 nm (UV-C and UV-B), the SA film’s light transmittance sharply rose, while the light transmittance of the SA-TA films in this range was 0%. This suggests that the SA-TA films exhibit excellent UV absorption ability and the addition of TA is more helpful to prevent UV light. This behavior was owed to the absorption of UV light by abundant aromatic groups and conjugation of phenol hydroxyl and carbonyl in the TA molecules [12,22,31,32]. From the obtained transmittance results, SA-TA films exhibited an excellent UV-shielding performance by completely absorbing the entire UVB and UVC region and absorbing the UVA in a certain degree. Therefore, the SA-TA film is a promising UV-barrier material that can prolong food product shelf life without significantly sacrificing transparency.

### 3.3. Color Parameter

Color is an important attribute of packaging films for affecting consumer acceptance. Therefore, the color parameters of the edible SA-TA films were characterized (Table 2). There was no significant difference in the films’ lightness (L*), indicating that TA addition did not affect the films’ brightness. In addition, the redness/greenness (a*) and yellowness/blueness (b*) values were close to 0, suggesting that all the films were transparent with little reddish and yellowish coloring. Regarding the color parameters of b*, the value increased significantly from 0.485 ± 0.0129 to 3.125 ± 0.01 with the incorporation of TA, meaning that the color of films changed to yellow. Furthermore, increased TA concentration in films led to significant increases in the total color difference (△E) and chroma (C*). The significant difference of these color parameters can be attributed to the brownish-yellow color of TA. These results are consistent with the film’s appearance, as shown in the photographs (Figure 4). The whiteness index (WI) of the films showed insignificant difference among all the films. It can be concluded that the incorporation of TA increased the films’ yellowish coloring without sacrificing their transparency, which can be observed by the naked eye. This phenomenon was similar to the effect of other polyphenol compounds on packaging films’ color reported in previous studies [12,27].

### 3.4. Water Vapor Permeability (WVP)

**Figure 5:**
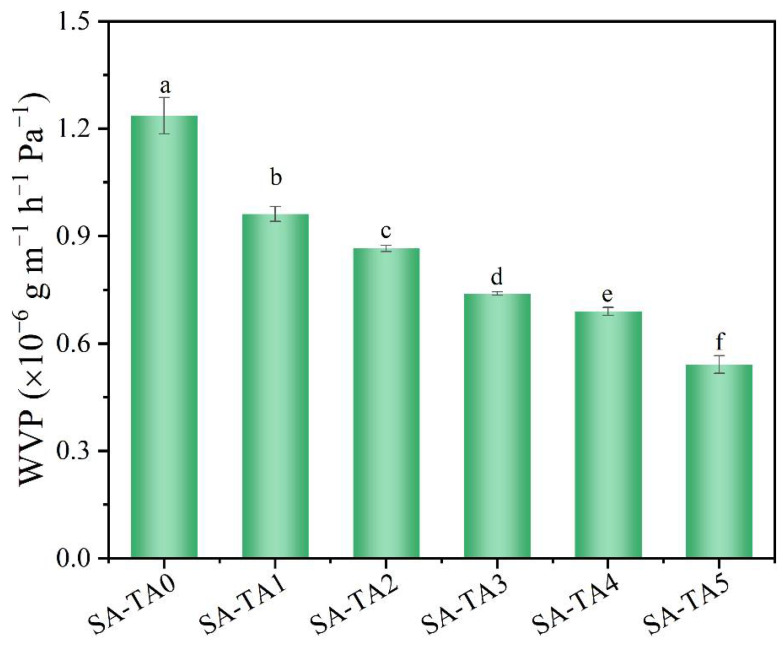
Water permeability of SA and SA-TA films. Different letters indicate significantly different groups (*p* < 0.05) based on ANOVA followed by Tukey test.

The capability to prevent moisture transfer from food products to the surrounding atmosphere is important for packaging films [33]. WVP is a vital property to evaluate the moisture barrier ability of films, and lower WVP is preferred since it can reduce the moisture loss of wrapped food products. According to Figure 5, the SA/TA composite films had better moisture barrier performance compared to SA film due to their lower WVP. It was proposed that a more compact internal network structure was formed between SA and TA due to the formation of hydrogen bonds, which resulted in a lower WVP [10]. Similar decrease in WVP was also observed in other polysaccharides films incorporated with tea polyphenols [10,12]. What is more, the films’ WVP decreased significantly from 1.24 × 10^−6^ g/m/h/Pa to 0.54 × 10^−6^ g/m/h/Pa with the TA concentration. This is owing to the exchange of water vapor being prevented by the stronger hydrogen bonds formed between TA and SA [10]. In addition, according to the results of FTIR, the numbers of intermolecular hydrogen bonds between TA and SA increased with TA concentration, which may reduce the hydrophilic groups’ availability in the film and lead to a reduction of hydrogen bonds between SA and water, thereby enhancing the films’ water permeability [34,35].

### 3.5. Mechanical Properties

**Figure 6:**
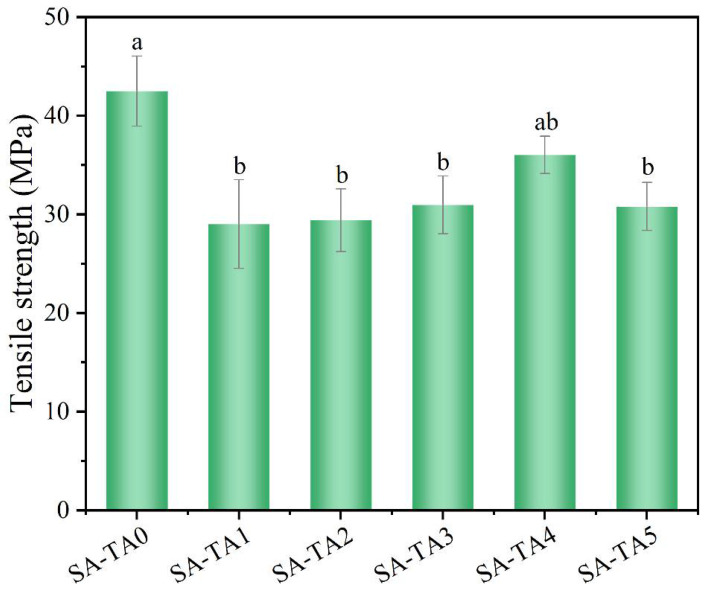
Tensile strength of SA and SA-TA films. Different letters indicate significantly different groups (*p* < 0.05) based on ANOVA followed by Tukey test.

Mechanical performances are of great importance for films’ service life. The tensile strength of SA film without TA was 42.5 MPa. However, the SA-TA composite films exhibited lower tensile strength (Figure 6). This was probably owing to the destructive force for SA film caused by the TA being stronger than the hydrogen bonding force between SA and TA [36]. As the TA concentration increased, the films’ tensile strength varied between 29.0 ± 4.5 MPa and 36.0 ± 1.9 MPa. The increase in tensile strength for SA-TA films was due to the stronger hydrogen bonding and chain entanglements between SA and TA at higher TA concentration. The slight decrease in tensile strength of SA-TA5 may be attributed to the formation of intramolecular hydrogen bonding interactions of TA caused by the high level of TA rather than intermolecular hydrogen bonding interactions between SA and TA [37]. However, we did not identify any significant differences in tensile strength of SA-TA films between the different formulations. This result was similar with other studies about SA-based films [26,27].

### 3.6. X-ray Diffraction (XRD)

**Figure 7:**
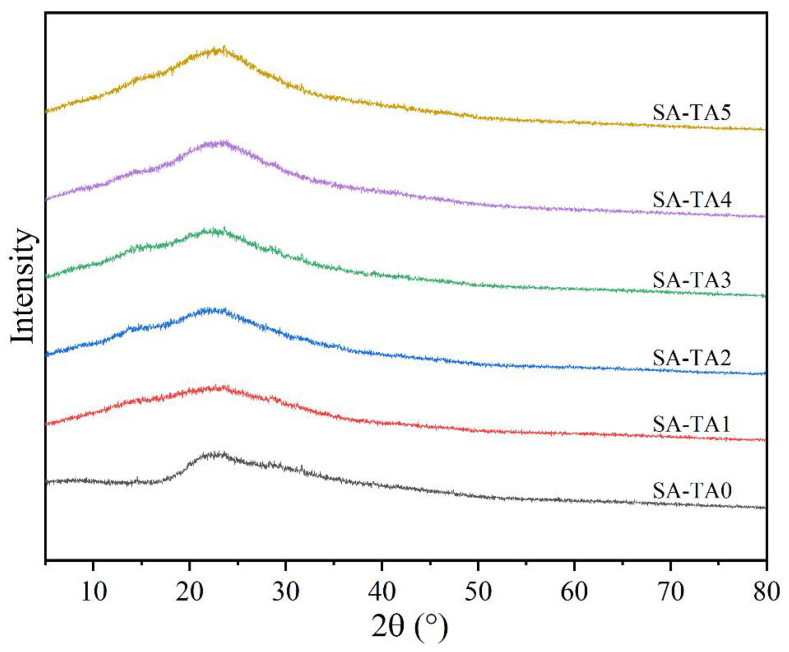
XRD pattern of SA and SA-TA films.

It was viewed from the XRD pattern that the SA film showed a broad and weak diffraction peak at 2θ = 22.4° (Figure 7), which indicated the low crystallinity of SA [26], and similar with other studies, the SA films showed broad peaks and amorphous characters [28]. Similar patterns were observed for all SA-TA films, suggesting TA had no influence on the films’ crystal structure. However, the diffraction peak of SA slightly shifted from 22.4° to 23.2°. This may be attributed to the formation of hydrogen bonds between TA and SA. In addition, the diffraction intensity of the crystalline peak showed an insignificant increase. This result suggested the formation of intermolecular interactions between the SA and TA chains [26]. Because the crystallization of SA stemmed from the intermolecular interactions between SA and TA molecular chains modulated by the hydrogen bonds [38], the incorporation of TA slightly increased the composite films’ crystallinity. The modulation effect caused by the incorporation of TA was confirmed in the FTIR spectra by the shift of wavenumber and vibration intensity changes of functional groups.

### 3.7. Thermal Stability Analysis

**Figure 8:**
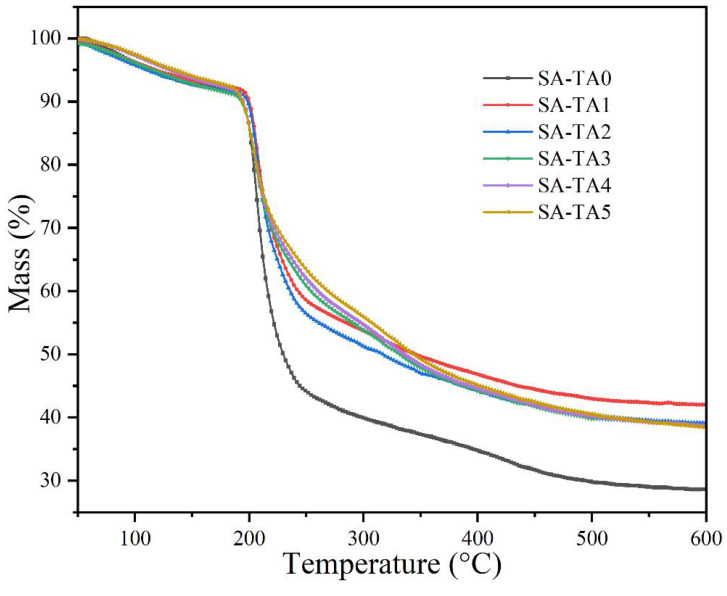
TGA curves of SA and SA-TA films.

The films’ thermal stability was evaluated by TGA (Figure 8). It suggested that all the SA-TA films underwent three stages of decomposition. The first decomposition stage took place before 160 °C with a weight loss about 6.5%. This was attributed to the water evaporation. The second decomposition stage occurred between 195 °C and 340 °C and it was the main decomposition stage. In this stage, the weight loss was attributed to the dehydration of the saccharide rings, decomposition and breaking of the main chain of the biopolymer. In addition, the de-crosslinking of network structures should also be considered [39]. In the second stage, some intermediate compounds may be formed, which was caused by the decarboxylation and esterification reactions [40]. At temperature higher than 340 °C, the weight loss can be attributed to further decomposition of the intermediate compounds.

The initial thermal degradation temperature (*T*_onset_) of the films from SA-TA0 to SA-TA5 were 198.1 °C, 200.6 °C, 200.5 °C, 196.2 °C, 195.3 °C, and 194.4 °C, respectively. It suggested that the incorporation of TA in the SA films slightly influence their thermal stability, and the *T_onset_* of the films increased firstly and then decreased with the incorporation of TA. The slight increase of *T_onset_* was probably owed to the strong interaction between SA and TA caused by the formation of hydrogen bonding [1], while the decrease of *T_onset_* may be owed to the extra intramolecular hydrogen bonding formed between TA [26]. Furthermore, the incorporation of TA decreased the mass loss rate and increased the residual mass of the SA film. This behavior suggested that the incorporation of TA improved the thermal stability of SA films clearly. Similar behavior was reported in other studies that the SA films’ thermal stability was enhanced by the addition of graphene oxide and hydrolyzed collagen [26,41].

### 3.8. Antioxidant and Antimicrobial Activity

**Figure 9:**
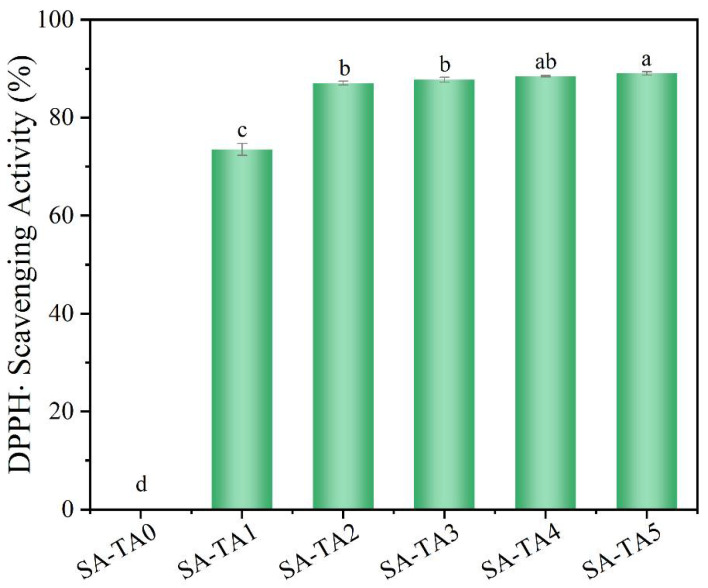
DPPH radical scavenging activity of SA and SA-TA films. Different letters indicate significantly different groups (*p* < 0.05) based on ANOVA followed by Tukey test.

Antioxidant activity, especially free radical scavenging activity, of food packaging films is a critical performance. Therefore, DPPH radical scavenging activity was measured to evaluate the films’ antioxidant activity (Figure 9). The SA film without TA showed no DPPH radical scavenging ability. However, the SA-T composite films’ radical scavenging capability was significantly improved. As the TA content in the SA-TA films increased, the composite films showed expected antioxidant activity and the radical scavenging activity reached to 89.2%. The enhanced antioxidant capability of the SA-TA films suggests that TA is an excellent antioxidant. The high radical scavenging activity of the TA was owed to its high content of polyphenolic hydroxyl groups that act as hydrogen donors and efficiently quench the free radicals [11,42,43]. Similar results have also been reported in other polysaccharide-based films that incorporated with polyphenol [1,6,12,25,43]. The excellent antioxidant activity performance indicates that the SA-TA films can be applied as promising packaging films in improving the food’s shelf life, especially for oxygen sensitive foods.

**Figure 10:**
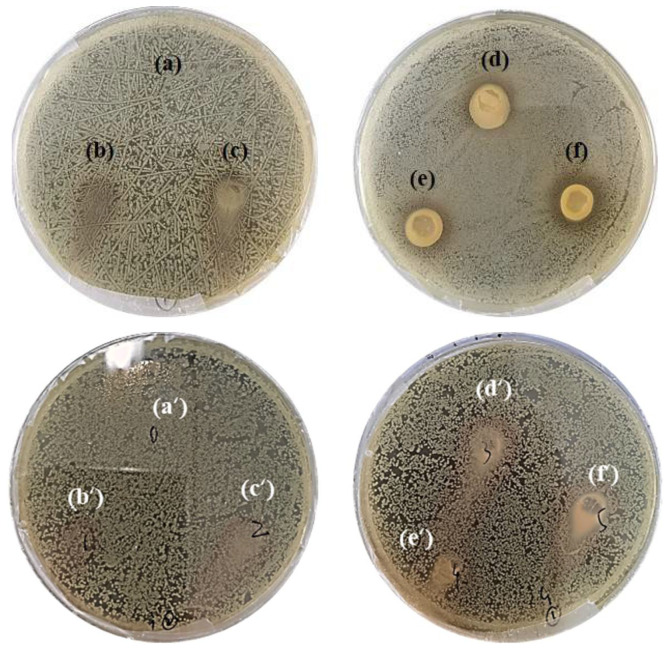
Antimicrobial effect against E. coil, (**a**) SA-TA 0, (**b**) SA-TA 1, (**c**) SA-TA 2, (**d**) SA-TA 3, (**e**) SA-TA 4, (**f**) SA-TA 5; and S. aureus, (**a’**) SA-TA 0, (**b’**) SA-TA 1, (**c’**) SA-TA 2, (**d’**) SA-TA 3, (**e’**) SA-TA 4, (**f’**) SA-TA 5.

The antimicrobial activity of SA-TA films against *E. coil* and *S. aureus* was evaluated by agar disk diffusion method (Figure 10). For the SA films without TA, both bacteria grew as normal, suggesting the SA films have no antimicrobial activity against *E. coil* and *S. aureus*. When the TA was incorporated into the SA films, the composite SA-TA films showed obvious inhibition effect against *E. coil*, suggesting that TA can inhibit the bacterial growth. Furthermore, the inhibition zones’ diameter significantly increased with the TA concentration. The inhibition effect of TA was owed to its ability to damage the bacterial cell membrane and eventually led to bacterial death by inducing leakage of intracellular components [1,11]. It is worth noting that the antimicrobial activity of SA-TA films against Gram-negative bacteria (*E. coil*) was greater than that against Gram-positive bacteria (*S. aureus*, there was no obvious inhibition effect can be observed). Gram-negative and Gram-positive bacteria differ in their cell wall structures, with peptidoglycan layer of the latter being much thicker, such structural differences might explain their different sensitivities toward the SA-TA films. However, our results about the antimicrobial activity against these two bacteria was different from other studies on polysaccharides/polyphenol composite films [4,11]. The relevant mechanism was unknown, and further research needs to be performed in the future.

## 4. Conclusions

Bioactive and edible SA-TA composite films were successfully prepared, and the impact of TA on the films’ optical, light barrier, water vapor permeability, antioxidant capability, and antimicrobial effect against *E. coil* and *S. aureus* was analyzed. With the incorporation of TA, the SA-TA films’ water vapor permeability significantly decreased, indicating the films’ water barrier ability was improved. This phenomenon was owed to the formation of hydrogen bonding between SA and TA, which was confirmed by the results of FTIR. The incorporation of TA significantly enhanced the SA-TA films’ UV-shielding ability and antioxidant capacity while increasing the films’ antibacterial effect on Gram bacteria, especially against *E. coil.* In addition, the TA concentration in the composite SA-TA films showed less impact on their transparency and mechanical properties. The improved properties caused by the incorporation of TA suggests that the SA-TA films should be a potential and green bioactive packaging material for food shelf-life extension.

## Figures and Tables

**Table 1:** Composition of the film forming solutions.

Sample ID	SA (g)	TA (g)	Glycerol (g)	Water (g)
SA-TA0	1.0	0	0.2	99
SA-TA1	1.0	0.2	0.2	99
SA-TA2	1.0	0.4	0.2	99
SA-TA3	1.0	0.6	0.2	99
SA-TA4	1.0	0.8	0.2	99
SA-TA5	1.0	1.0	0.2	99

**Table 2:** Color parameters including L*, a*, b*, ΔE*, WI, and C* of films.

Film	L*	a*	b*	ΔE*	WI	C*
SA-TA0	41.878 ± 0.096	−0.06 ^ab^ ± 0.0115	−0.485 ^f^ ± 0.0129	—	41.875 ± 0.096	0.489 ^f^ ± 0.0116
SA-TA1	41.738 ± 0.129	0.105 ^a^ ± 0.0129	0.538 ^e^ ± 0.0171	1.056 ^e^ ± 0.0307	41.735 ± 0.129	0.548 ^ef^ ± 0.0182
SA-TA2	41.538 ± 0.350	−0.00575 ^ab^ ± 0.00675	0.685 ^d^ ± 0.0465	1.262 ^d^ ± 0.0519	41.533 ± 0.349	0.685 ^d^ ± 0.0465
SA-TA3	41.82 ± 0.502	−0.12 ^ab^ ± 0.00816	1.46 ^c^ ± 0.102	2.000 ^c^ ± 0.0861	41.801 ± 0.500	1.465 ^c^ ± 0.102
SA-TA4	41.872 ± 0.310	−0.175 ^b^ ± 0.01	2.392 ^b^ ± 0.0613	2.896 ^b^ ± 0.0620	41.823 ± 0.312	2.398 ^b^ ± 0.0609
SA-TA5	41.618 ± 0.314	−0.238 ^b^ ± 0.005	3.125 ^a^ ± 0.010	3.639 ^a^ ± 0.0143	41.533 ± 0.314	3.134 ^a^ ± 0.0101

Values are presented as mean ± standard deviation. Different letters in the same column indicate significantly different groups (*p* < 0.05) based on ANOVA followed by Tukey test.

## Data Availability

Data is contained within the article.
